# Nematode homologs of the sour taste receptor Otopetrin1 are evolutionarily conserved acid-sensitive proton channels

**DOI:** 10.3389/fcell.2023.1133890

**Published:** 2023-01-26

**Authors:** Shitian Li, Umar Al-Sheikh, Yili Chen, Lijun Kang

**Affiliations:** ^1^ Department of Neurobiology and Department of Neurosurgery of the Fourth Affiliated Hospital, Zhejiang University School of Medicine, Hangzhou, China; ^2^ Liangzhu Laboratory, MOE Frontier Science Center for Brain Science and Brain-machine Integration, State Key Laboratory of Brain-machine Intelligence, Zhejiang University, Hangzhou, China; ^3^ NHC and CAMS Key Laboratory of Medical Neurobiology, School of Medicine, Zhejiang University, Hangzhou, Zhejiang, China

**Keywords:** acid sensation, Caenorhabditis elegans, sensory receptors, otopetrin channels, calcium imaging

## Abstract

Numerous taste receptors and related molecules have been identified in vertebrates and invertebrates. Otopetrin1 has recently been identified as mammalian sour taste receptor which is essential for acid sensation. However, whether other Otopetrin proteins are involved in PH-sensing remains unknown. In *C. elegans*, there are eight *otopetrin* homologous genes but their expression patterns and functions have not been reported so far. Through heterologous expression in HEK293T cells, we found that ceOTOP1a can be activated by acid in NMDG^+^ solution without conventional cations, which generated inward currents and can be blocked by zinc ions. Moreover, we found that Otopetrin channels are widely expressed in numerous tissues, especially in sensory neurons in the nematode. These results suggest that the biophysical characteristics of the Otopetrin channels in nematodes are generally conserved. However, a series of single gene mutations of *otopetrins,* which were constructed by CRISPR-Cas9 method, did not affect either calcium responses in ASH polymodal sensory neurons to acid stimulation or acid avoidance behaviors, suggesting that Otopetrin channels might have diverse functions among species. This study reveals that nematode Otopetrins are evolutionarily conserved acid-sensitive proton channels, and provides a framework for further revealing the function and mechanisms of Otopetrin channels in both invertebrates and vertebrates.

## Introduction

Otopetrins are recently identified as proton-selective ion channels (Tu et al., 2018). There are numerous types of ion channels which selectively permeate the flow of sodium, potassium, calcium, or chloride ions (Gouaux and Mackinnon, 2005; Catterall et al., 2017), but the number of proton channels are relatively small in nature (Decoursey, 2003). The Matrix-2 (M2) proteins in the influenza A virus permeate protons into the interior of virions (Pinto et al., 1992). Prior to the discovery of Otopetrin channels, voltage-gated Hv1 was the only known proton channel in eukaryotes (DeCoursey, 1991; Ramsey et al., 2006; Sasaki et al., 2006), which functions in many physiological and pathophysiological processes such as regulating cellular pH in response to depolarization and maintaining pH in human neutrophils during phagocytosis (Morgan et al., 2009). Another recently discovered proton-selective channel in mammals, TMEM175, is proton-activated and expressed on lysosomal membranes mediating protonic fluid to maintain pH homeostasis (Hu et al., 2022).

Otopetrin1 (OTOP1) was initially uncovered by its role in the vestibular system of mice and zebrafish (Hurle et al., 2003; de Caprona et al., 2004; Hughes et al., 2004; Söllner et al., 2004). In the mammalian inner ear, otoliths are hard, calcium carbonate structures that sense gravity and linear acceleration (Hu et al., 2022). The zebrafish Otopetrin, zeOTOP1 is required for otolith formation (Hughes et al., 2004). As such, the protein was named as Otopetrin for its influence on the development of murine otolith. Mutant alleles, *tlt* and *mlh*, of *otop1* cause abnormalities in the murine vestibular system (Hurle et al., 2003; Hughes et al., 2007), disrupting the interaction of *otop1* with other proteins and membrane trafficking in the vestibular support cells (Kim et al., 2011). In addition, mutant mice exhibit abnormalities in forced swimming tests due to the degeneration of otolith (Ornitz et al., 1998). In sea urchins, Otopetrin functions as a proton channel to promote clearance of protons during calcium carbonate production in primary mesenchymal cells (Chang et al., 2021).

The *otopetrin* gene family is evolutionarily conserved from *Caenorhabditis elegans* to humans (Hurle et al., 2003; Hughes et al., 2008). There are three murine *otop1* homologous genes in the *Drosophila melanogaster* genome (Hurle et al., 2003; Hughes et al., 2008) and eight *otopetrin* genes in *C. elegans* (Hurle et al., 2011). The murine Otopetrin family is widely expressed in various tissues (Tu et al., 2018), suggesting their involvement in physiological processes.

Heterologous expression of *otopetrin* cDNA from mouse, *Drosophila*, *Zebrafish*, and *Xenopus* in HEK293 cells induced a large inward proton current responding to extracellular acidification which can be blocked by Zn^2+^ (Tu et al., 2018; Chen et al., 2019; Saotome et al., 2019; Mi et al., 2021). In addition, Otopetrin channels mediate the flow of protons at resting potential to permeate the cytoplasm. The Otopetrin family and other types of ion channels are not structurally similar based on Cryo-EM structural analysis (Hughes et al., 2008; Chen et al., 2019; Saotome et al., 2019). Amino acid mutations in the predicted proton permeability sequences can significantly disturb the recorded proton channel currents (Chen et al., 2019; Saotome et al., 2019).

Over the past decades, numerous taste receptors and related molecules have been identified in vertebrates and invertebrates (Liman et al., 2014). Taste receptor cells respond to different taste stimuli which include sweet, salty, bitter, sour and umami tastes (Chandrashekar et al., 2006). Subsequent studies found that the receptor for salty taste is the sodium ion-permeable ENaC channel (Chandrashekar et al., 2010; Nomura et al., 2020). The discovery of sour taste receptors was relatively delayed until the Otopetrin family was identified as novel proton channels (Tu et al., 2018), then OTOP1 was confirmed to be the sour taste receptor after a series of experimentations (Liman and Kinnamon, 2021; Turner and Liman, 2022). Notably, in the study of sour taste there is a proton-sensitive conductance in taste receptor cells which is different from other taste stimuli (Chang et al., 2010). OTOP1 is specifically expressed in the sour taste receptor cells of mice, and its mutation inhibit the proton current generated by acid stimulation in sour taste receptor cells (Tu et al., 2018). Furthermore, *Otop1* knockout mice have significantly reduced taste receptor neural responses to both hydrochloric acid and citric acid stimulation (Teng et al., 2019) indicating that OTOP1 is definitely the sour receptor in mice (Zhang et al., 2019). In addition, the Otopetrin-like protein OtopLA is also the sour taste receptor in *Drosophila* (Ganguly et al., 2021; Mi et al., 2021), suggesting that the Otopetrin1 may function as the universal sour taste receptors across species (Frank et al., 2022). However, it remains unknown whether other Otopetrin channels are required for acid sensation, or they are involved in avoidance behaviors in response to aversive acidic pH.

*C. elegans* can detect a variety of stimuli including chemical stimuli like acidic pH (Bargmann, 2006; Iliff and Xu, 2020). The ASH sensory neurons sense acidic stimulation and mediate acid avoidance behavior (Hilliard et al., 2004; Wang et al., 2016). The nematode consists of eight *otopetrin* genes. In this study, we explored the biophysical characteristics, the expression patterns and the functions of *C. elegans* Otopetrins.

## Materials and methods

### Worm cultivation

Worms were cultivated at 20°C on NGM plates seeded with OP50 *E. Coli*. The well-fed adult hermaphrodites were used in all experiments. Bristol N2 and *osm-9(ky10)* were obtained from the *Caenorhabditis Genetics Center*. *KanIS8 [Psra-6::GCaMP5.0 + Psra-6::mCherry + Plin-44::GFP]* stain was served as the control strain in recording of acid-evoked calcium responses and currents.

### Molecular biology

Promoter fragments were designed and amplified from *C. elegans* N2 strain and then cloned into *pBS77::mCherry* vector using the In-Fusion PCR Cloning Kit (TaKaRa Inc.). The promoters used are as follow: *otpl-1*: 1,164 bp upstream of *otpl-1* start codon; *otpl-2*: 2,506 bp upstream of *otpl-2* start codon; *otpl-3*: −814 bp∼ + 132 bp; *otpl-4*: −614 bp−1 bp; *otpl-5*: −1,036 bp801 bp; *otpl-6*: −1,518 bp−1 bp; *otpl-7*: −2,502 bp−1 bp; and *otpl-8*: 2,698 bp−1 bp. Full-length *Otop1a* cDNA was amplified by PCR from template obtained from all-worm RNA reverse transcription and cloned into pEFGP-C3 vector.

### Confocal imaging

Worms were immobilized using 200 mM sodium azide (NaN_3_). Images were captured by an inverted Olympus FV1000 laser scanning confocal microscope with filter setting for both GFP and mCherry.

### Generation of otopetrin mutants by CRISPR-Cas9

Knockout *otopetrin* strains were obtained by CRISPR-Cas9 referring to Friedland’s method (Friedland et al., 2013). Briefly, exons located in front of the *otopetrin* genes were chosen as targets. Based on the selected sequences, specific guide RNAs (sgRNAs) were predicted and designed. Nematodes were then genotyped after microinjection of the plasmid to obtain the desired mutant strains of the target genes. CRISPR-Cas9 plasmids with *eft-3* promoter were used to finally obtain whole worm knockout mutants. CRISPR-Cas9 knockout plasmids of *dpy-10* were co-injected to produce chubby phenotypes as a pick-out marker and a system control. The *otopetrin* mutants selected in this study were all frameshift mutations. The sgRNAs for each *otopetrin* mutant and PCR primers used to genotype each *otopetrin* mutant are available in the Supplementary Material as TablesS1, S2, respectively.

### Calcium imaging

Day 1 adult hermaphrodites were immersed in bath solution and glued on a glass coverslip. The bath solution contained 145 mM NaCl, 5 mM KCl, 1 mM MgCl_2_, 2 mM CaCl_2_, 20 mM glucose, 10 mM HEPES (pH 7.3 with NaOH). The acid stimulation solution contained 145 mM NaCl, 5 mM KCl, 1 mM MgCl_2_, 2 mM CaCl_2_, 20 mM glucose, 10 mM MES, which was adjusted to pH 2.5, 3 or 4 with HCl. GCaMP5.0 was used as the calcium indicator and images were acquired using an Olympus microscope (IX71) under a × 40 objective lens coupled with an Andor DL-604M EMCCD camera. Data were collected using the Micro-Manager software. GCaMP5.0 was excited by a ThorLabs blue light (460–480 nm) LED lamp. The average GCaMP5.0 signal in the first 10 s before stimulation was set as F0 and △F/F0 was calculated for each data point. All image stacks were analyzed using the Image-J software (Cheng et al., 2022).

### Behavioral assays

A drop test was used to detect the avoidance behavior of nematodes. Day 1 adult hermaphrodites were transferred onto unseeded NGM plates. Using a micropipette, an acid stimulation solution (pH 3) droplet was delivered near the tail, flowed along the body and finally to the nose tip of nematode. Reversal behavior was defined as going backward for more than two body bends and less than 3 s responses. Worms presenting avoidance response was counted as 1, and no response was counted as 0. Each *otopetrin* mutant was tested on five different days, with approximately 30 worms tested each day. Wild-type and *osm-9* mutants were employed as positive and negative controls. The avoidance response ratio of all the worms tested from each strain was calculated and statistically analyzed by One-way analysis of variance (ANOVA) and Dunnett’s test.

### Transfection in HEK293T cells

Full-length *ceOTOP1a* cDNA was cloned into pEFGP-C3 vector. EGFP-ceOTOP1a was co-transfected with EGFP-empty (5:1) into HEK293T cells using Lipofectamine 2000 Transfection Reagent. Cells were detached by trypsin-EDTA 1 day before patch clamp recording and transferred onto coverslips coated with poly-lysine.

### Patch-clamp electrophysiology

For HEK293T cells recording, the extracellular solution contained 160 mM NMDG, 2 mM CaCl_2_, 10 mM HEPES (pH 7.4 with HCl). Acidic solution contained 160 mM NMDG, 2 mM CaCl_2_, 10 mM MES (pH 4.5 with HCl), 1 mM ZnCl_2_ was added additionally for Zn^2+^-sensitivity test. The pipette solution contained 120 mM Cs-aspartate, 15 mM CsCl, 2 mM Mg-ATP, 5 mM EGTA, 2.4 mM CaCl_2_, 10 mM HEPES (pH 7.3, 290 mOsm).

For ASH *in vivo* recordings, the bath solution (NaCl, pH 7) contained 145 mM NaCl, 2.5 mM KCl, 5 mM CaCl_2_, 1 mM MgCl_2_, 20 mM glucose, 10 mM HEPES (pH 7.3, 325–335 mOsm). Extracellular solution (NMDG, pH 7) contained 145 mM NMDG, 2.5 mM KCl, 5 mM CaCl_2_, 1 mM MgCl_2_, 20 mM glucose, 10 mM HEPES (pH 7.3, 325–335 mOsm). Extracellular solution (NMDG, pH 3) contained 145 mM NMDG, 2.5 mM KCl, 5 mM CaCl_2_, 1 mM MgCl_2_, 20 mM glucose, 10 mM MES (pH 3, 325–335 mOsm), 1 mM ZnCl_2_ was added additionally for Zn^2+^-sensitivity test. The pipette solution contained 145 mM KCl, 5 mM MgCl_2_, 5 mM EGTA, 0.25 mM CaCl_2_, 10 mM HEPES, 10 mM glucose, 5 mM Na_2_ATP, 0.5 mM Na_2_GTP (pH 7.2, 315–325 mOsm).

Electrophysiological recordings were performed on an Olympus microscope (BX51WI) with an EPC-10 amplifier and Patchmaster software (HEKA). Day 2 adult worms were glued on the surface of sylgard-coated coverslips using cyanoacrylate-based glue. The soma of the ASH neurons were exposed with a dorsolateral incision made by a sharp glass pipettes. The membrane potential was clamped at −60 mV.

### Statistical analysis

Data was analyzed using GraphPad Prism 5 and Igor Pro5.0 software (Wavemetrics). Error bars are presented as mean ± SEM.

## Results

### Expression patterns of nematode otopetrin gene family

**FIGURE 1:**
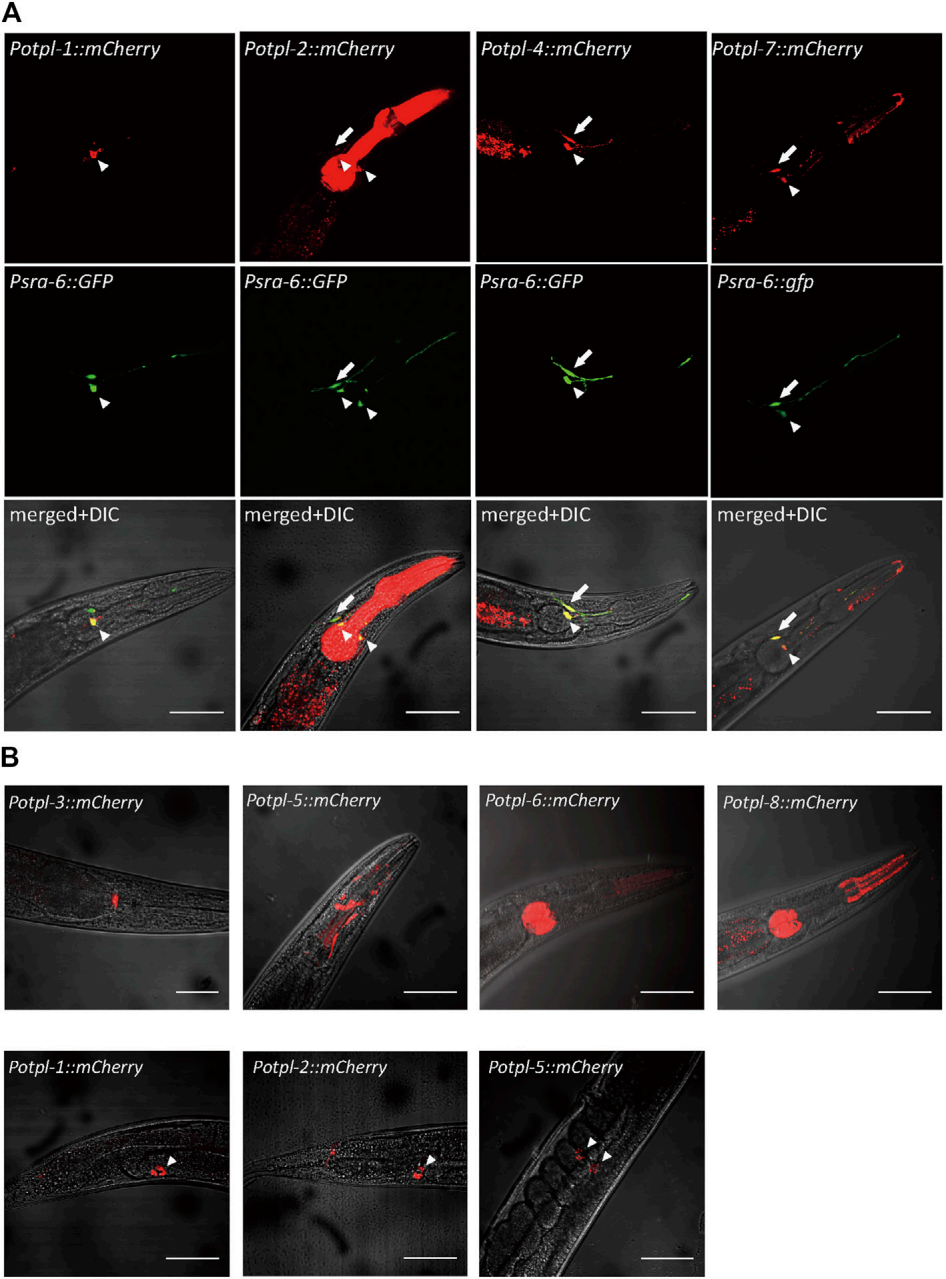
Expression patterns of *C. elegans otopetrins*. **(A)** Confocal images show four *otopetrin* gene co-located with head sensory neurons ASH and ASI neurons. *Psra-6::GFP* labeled ASH and ASI neurons with green fluorescent proteins. Arrowheads indicate ASI neurons, and arrows indicate ASH neurons. Scale bar, 50 μm. **(B)** The expression of *otopetrin* gene family in the nematode pharynx and head (upper) and in coelomocytes (lower). Scale bar, 50 μm.

We firstly sought to explore the expression patterns of the *otopetrin* genes, and constructed exogenous expression lines by microinjection. Using the promoters of *otopetrins* fused with mCherry, the expression patterns of all eight *otopetrin* genes in *C. elegans* were observed. The *otopetrin* genes *otpl-1*, *otpl-2*, *otpl-4*, and *otpl-7* were expressed in sensory neurons in the head of the worms clearly co-localized with *Psra-6::GFP,* which marks chemosensory neurons ASH and ASI (Figure 1A). Four genes (*otpl-1*, *otpl-2*, *otpl-4*, and *otpl-7*) showed strong expression in ASI neurons, and *otpl-2*, *otpl-4*, and *otpl-7* were also expressed in ASH neurons. The explicit expression of the *otopetrin* gene family in head sensory neurons suggests its underlying function might be related to acid sensation.

In the pharynx, *otpl-2, otpl-5, otpl-6*, and *otpl-8* were strongly expressed in different parts, and *otpl-3* was observed at the junction of the pharynx and gut (Figure 1B). Further, *otpl-5* was observed in the head and *otpl-1*, *otpl-2*, and *otpl-5* were expressed in coelomocytes (Figure 1B). Several *otopetrin* genes were co-expressed in same cell, suggesting they may function cooperatively or redundantly.

### *ceOTOP1a* mediates a zinc-sensitive acid-responsive current

**FIGURE 2:**
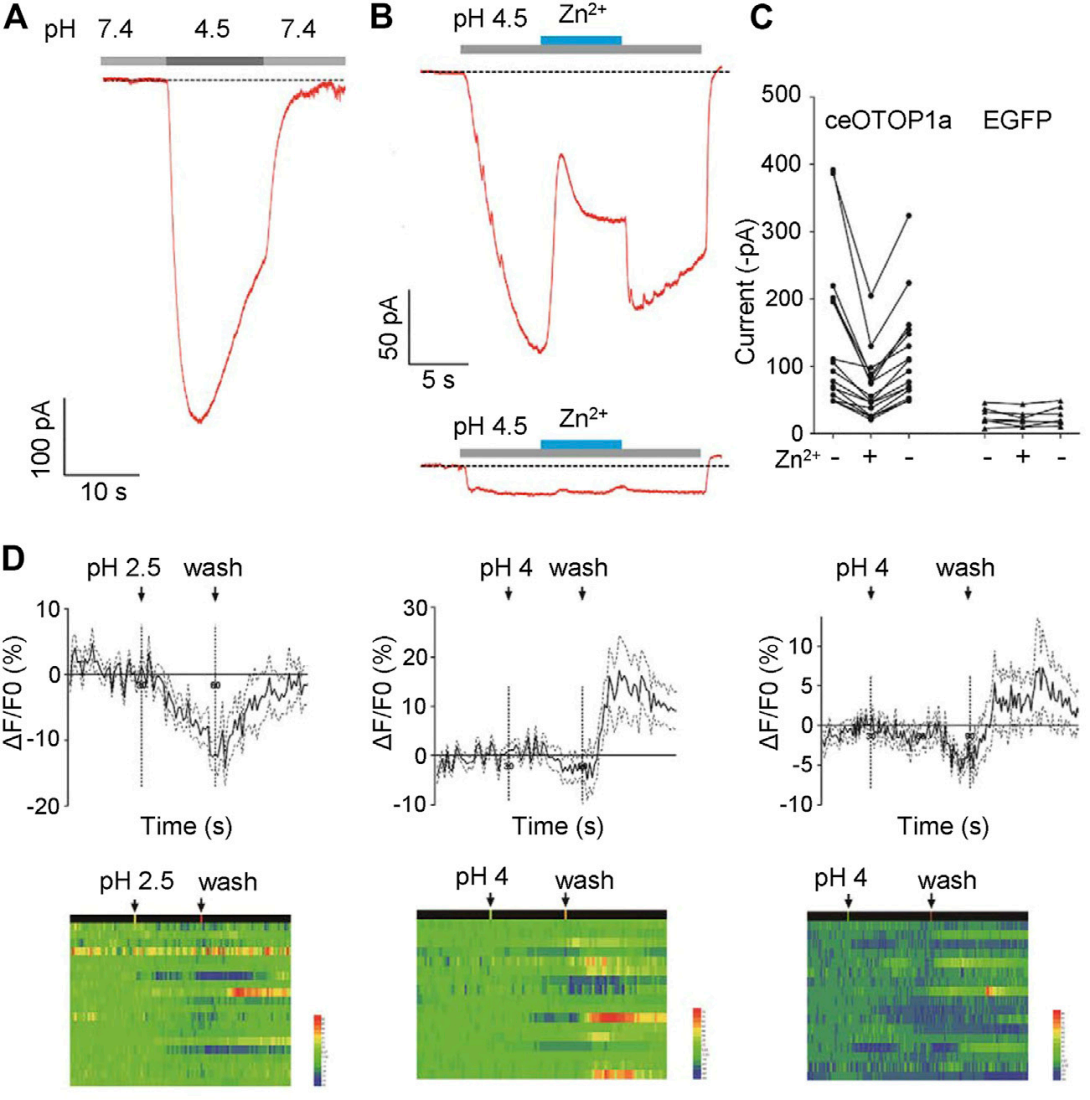
CeOTOP1a heterologous expression in HEK273T cells shows zinc ion-sensitive acid-responsive currents. **(A)** Acid-evoked currents were recorded by whole-cell patch clamp recording in HEK273T cells heterologously expressing nematode ceOTOP1a. The membrane potential was clamped at −80 mV. **(B)** Acid-evoked currents can be reversible by 1 mM Zn^2+^. The gravity perfusion system was used, switching channels to replace the solution. Membrane potential was clamped at −80 mV. Acid stimulation (grey), Zn^2+^ application (blue). **(C)** Statistics of the current amplitude. Membrane potential was clamped at −80 mV. **(D)** Acid stimulation does not induce significant calcium responses in ASI neurons.

Proton currents sensitive to zinc ions were recorded when the *otopetrin* gene family of mice, zebrafish, and chicken were heterologously expressed in HEK293 cells (Tu et al., 2018; Chen et al., 2019; Saotome et al., 2019; Mi et al., 2021). To explore the characteristics of *C. elegans* Otopetrin proteins, we heterologously expressed ceOTOP1a in HEK293T cell line. Full-length cDNA of worm OTOP1a was amplified and cloned into pEGFP-C3 vector. Cells marked by EGFP were chosen for patch clamp recording. Extracellular solutions were NMDG^+^-based, pH of the bath solution was 7.4 and pH of the acid stimulation solution was adjusted to 4.5. Large inward currents were recorded when the acid stimulation solution was given by a gravity perfusion system toward the cells (Figures 2A–C).

In order to determine whether the acid-responsive current is sensitive to zinc ions, ZnCl_2_ was added to the acid-stimulated external solution, and the final concentration of zinc ion was 1 mM. We found that the acid activated current was reversibly inhibited by zinc ions (Figures 2B, C). These results show that ceOTOP1a is a zinc-sensitive proton channel, suggesting that the nematode *otopetrin* gene is evolutionarily conserved with other species.

### Acid stimulation does not induce significant calcium response in ASI neurons

Given that *otopetrin* genes are expressed in ASI sensory neurons, we explore whether ASI neurons evoke calcium responses to acid stimulation. Stimuli were delivered in three different patterns; pH 2.5 for 30 s, pH 4 for 30 s, and pH 4 for 60 s. Under each stimulus, no significant calcium response was recorded during the time course (Figure 2D). Only a wash out response of about 10% was recorded, which was very weak compared to the response amplitude of ASI neurons to external stimuli such as Cu^2+^, suggesting that ASI may play a minor role in acid sensation.

### Acid stimulation to *otopetrin* single-gene mutants does not affect the calcium response of ASH neurons

**FIGURE 3:**
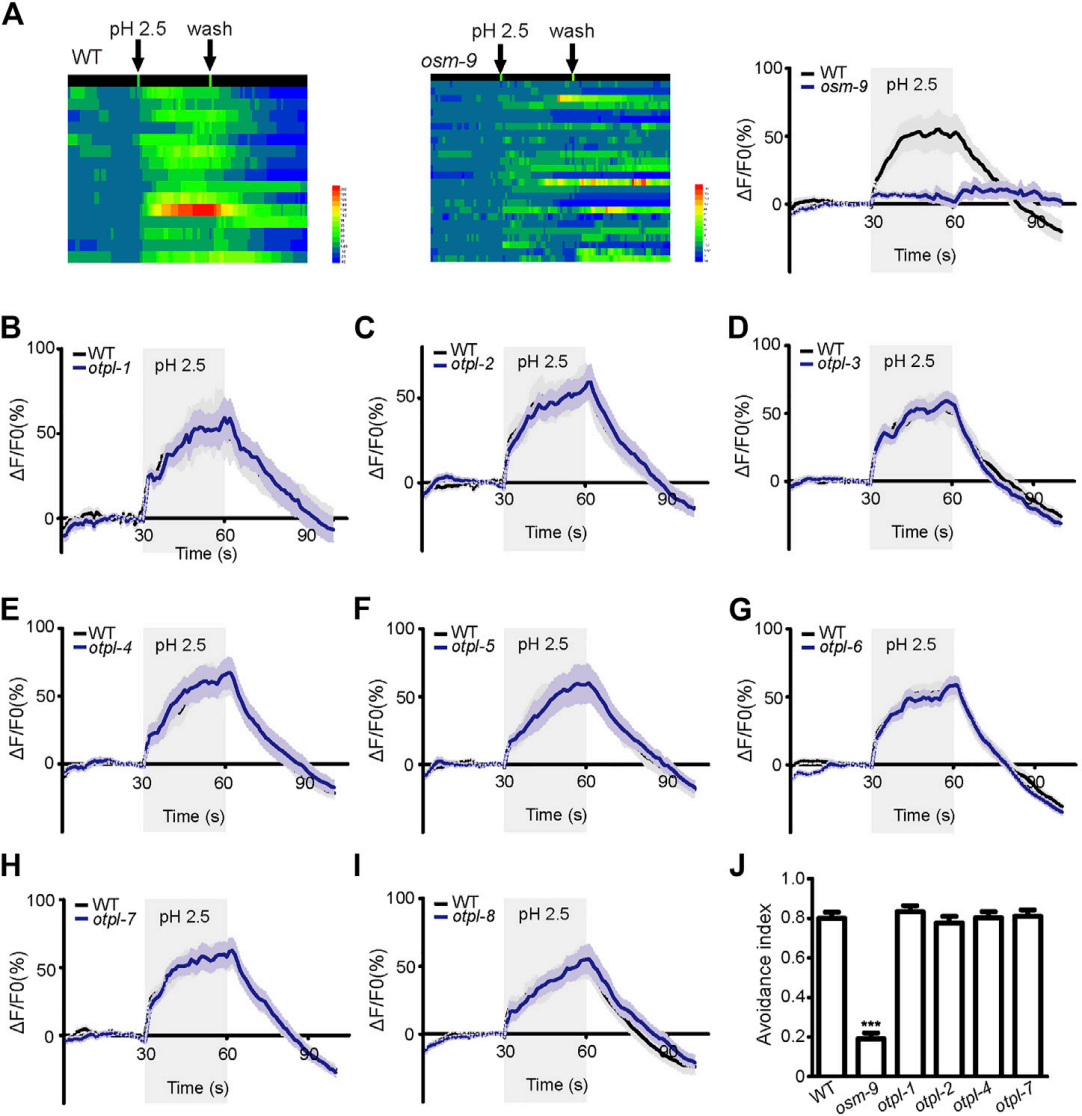
*Otopetrin* single gene mutations do not affect calcium responses of ASH neurons to acid stimulation and acid avoidance behavior in nematodes **(A)** ASH neurons were able to induce calcium responses to acid stimulation, *osm-9* mutant was used as a negative control. **(B–I)** Calcium responses of ASH neurons to acid solution stimulation in all eight *otopetrin* single mutants, which were not significantly different from WT. **(J)** The mutant of four ASH neuron-expressing *otopetrin* genes did not affect acid avoidance behavior of nematodes. The number tested of each strain were 160 (WT), 173 (*osm-9*), 150 (*otpl-1*), 152 (*otpl-2*), 162 (*otpl-4*), and 142 (*otpl-7*). One-way ANOVA, Dunnett’s test, ***p <0.001.

ASH neurons displayed robust calcium responses to acid stimulation when a pH 2.5 stimulus of 30 s duration was delivered (Figure 3A). The average amplitude of the response is about 50%. The pattern of the superimposed curve was generally that the amplitude increased after dosing, then reached a plateau, and the response amplitude decreased rapidly after elution (Figure 3A). The *osm-9* mutant strain showed almost no obvious calcium response to acid stimulation, which was used as a negative control (Figure 3A).

We then used CRISPR-Cas9 system to edit *otopetrin* genes and obtained single-gene knockout nematode mutants. *Otopetrin* mutants finally selected are all frameshift mutations. All *otopetrin* knockout mutants did not show significant developmental differences from WT.

Each mutant of the eight genes of the *otopetrin* family did not significantly affect acid stimulation-evoked calcium increases in ASH (Figures 3B–I). Given that only strains of single-gene mutants were tested, it is unclear whether multiple *otopetrins* mediate the calcium response of ASH to acid stimulation simultaneously.

### *Otopetrin* knockout mutants do not affect acid avoidance behavior in nematodes

Previous studies have showed that OTOP1 is the sour taste receptor in mice (Zhang et al., 2019) and OtopLA is the sour taste receptor in *Drosophila* (Ganguly et al., 2021; Mi et al., 2021). The functions of Otopetrins are highly consistent in various species, suggesting that Otopetrin1 may function as the universal sour taste receptor (Frank et al., 2022). In *C. elegans*, ASH neurons can sense acid stimulation and mediate avoidance response behaviors (Hilliard et al., 2004), which are impaired in *osm-9* mutants (Wang et al., 2016). According to the expression patterns, four *otopetrin* genes (*otpl-1*, *otpl-2*, *otpl-4*, and *otpl-7*) were co-localized within ASH sensory neurons, then we tested whether mutants of these *otopetrin* genes affect acid avoidance behavior.

A pH 3 bath solution droplet was delivered using a micropipette near the tail, flowed along the body and finally to the nose tip of the worm. When the acidic solution is in contact with the nose tip, the worm reversed—avoidance behavior. Worm presenting avoidance response with 3 s was counted as 1, and no response was counted as 0. WT and *osm-9* mutant strains were employed as positive and negative controls, respectively. The response of *osm-9* to pH 3 stimulation was significantly attenuated, whereas each mutant of the four *otopetrin* genes exhibited normal avoidance behaviors to acid stimulation similar to that of WT (Figure 3J).

### Single gene mutations of *otopetrins* do not influence the acid-evoked current of ASH neurons in nematodes

**FIGURE 4:**
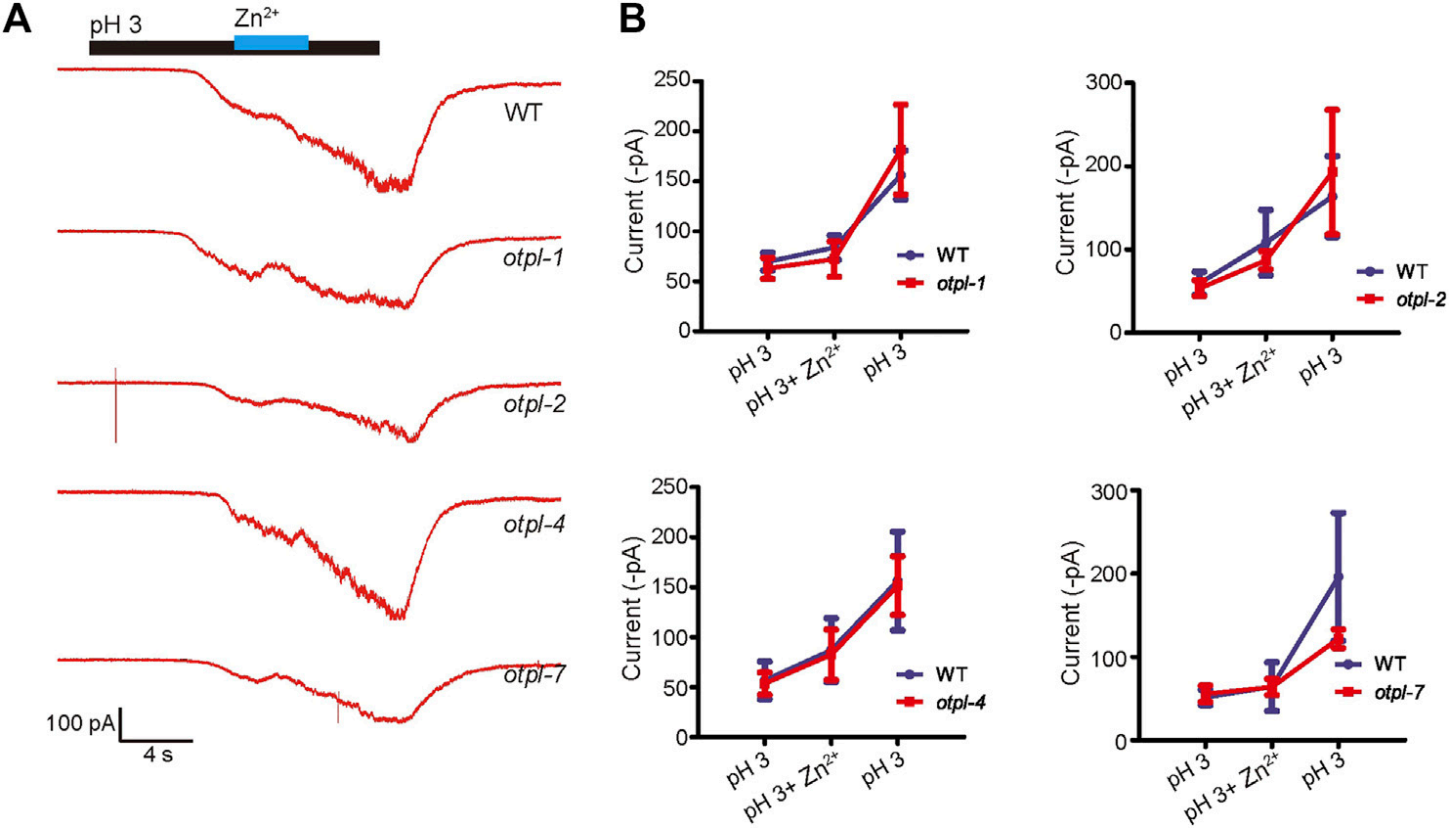
Single-gene *otopetrin* mutants do not affect *in vivo* acid-responsive currents of ASH neurons. **(A)** *In vivo* recording of ASH neurons in *C. elegans*, acid-sensing currents were not blocked by 1 mM Zn^2+^. The cation in solution is NMDG^+^. Gravity perfusion system was used to deliver acid stimulation, switching channels to replace the solution. Membrane potential was clamped at −60 mV. **(B)** Statistical values of the current. The membrane potential was clamped at −60 mV.

Since ASH neurons are sensitive to acidic stimulation, we then checked whether *otopetrin* mutants affect the acid-evoked currents of ASH recorded by *in vivo* patch clamp recordings. The pH 3-activated currents of ASH neurons were recorded, and 1 mM Zn^2+^ did not significantly inhibit the acid-sensing inward current (Figure 4A). Moreover, the current amplitudes from the *otopetrin* mutants (*otpl-1*, *otpl-2*, *otpl-4*, and *otpl-7*) were not significantly different from WT (Figure 4B). Single gene mutations of *otopetrins* did not affect the currents of ASH neurons in response to acidic stimulation, suggesting that multiple Otopetrins may mediate the acid-evoked currents of ASH simultaneously, or the acid-evoked currents of ASH are conducted by other molecules.

## Discussion

Similar to other species, when ceOTOP1a is heterologously expressed in HEK293T cells, a sodium-independent acid-responsive current blocked by zinc ions can be recorded responding to extracellular acidification. The nematode ceOTOP1a exhibits common electrophysiological properties of the Otopetrin channel family, indicating that the *C. elegans* Otopetrins are functionally conserved as proton channels.

Expression patterns indicate that *C. elegans otopetrins* are located in sensory neurons and many tissues. Among them, there is exact expression in ASI and ASH neurons, suggesting the potential function of Otopetrin as the sensory receptor. ASI did not show significant calcium responses to strong acid stimulation. We mainly tested the role of *otopetrin* mutants in ASH sensory neurons. *Otopetrin* gene knockout does not affect acid avoidance behavior in nematodes, all mutants of the *otopetrin* family still had robust calcium response to acid stimulation, and the *otopetrin* mutation did not affects acid activated currents of ASH. These data showed that single-gene mutants of the *otopetrin* family did not affect the response of nematodes to acid stimulation. Given there are eight *otopetrin* genes in nematodes, it is still unclear whether the polygenic mutations of *otopetrins* affect the acid response of nematodes.

The biological function of *otopetrin* gene in *C. elegans* and other species still to be discovered. Because of the widespread tissue expression, and the characteristics of Otopetrin channel are conserved. The follow-up topics should focus on screening and discovering the physiological functions of various *otopetrin* genes on various tissue cells based on the nematode system, and exploring their roles and regulatory mechanisms. It will lay a valuable scientific foundation for the discovery of the physiological function and pharmacological mechanism of mammalian Otopetrin proteins.

## Data Availability

The original contributions presented in the study are included in the article/Supplementary Material, further inquiries can be directed to the corresponding authors.
