# Supplementary material for: Nematode homologs of the sour taste receptor Otopetrin1 are evolutionarily conserved acid-sensitive proton channels
Source: Front Cell Dev Biol. 2023 Jan 26;11:1133890. doi: 10.3389/fcell.2023.1133890 (PMC9909269; doi:10.3389/fcell.2023.1133890)
Supplement: Supplementary file 1 [file Table1.DOCX]

**Supplementary Material**

**Supplementary Material**

**Table S1 to S2**

**Supplementary Material**

**CRISPR-Cas9**

*otpl-1*: 221-222bp (---AG) in spliced sequences of isoform A were changed into (CTCA-), totally 2bp insertion in the second exon of isoform A.

*otpl-2*: 299-308bp in spliced sequences were deleted, 10bp deletion (CCTTGCACAG) in the second exon.

*otpl-3*: 117-132bp (TTCAG--ATGCGTG-GTCC) in spliced sequences were changed into (---AGGTAT-C-TGAG--A), totally 4bp deletion in the first exon.

*otpl-4*: 63-66bp in spliced sequences were deleted, 4bp deletion (ATTA) in the second exon.

*otpl-5*: 86-111bp in spliced sequences were deleted, 26bp deletion (CAGACGGGTTCAGTAGCCATGACGAT) in the second exon.

*otpl-6*: 55-67bp in spliced sequences were deleted, 13bp deletion (GATGTTACTGTCC) in the second exon.

*otpl-7*: 102-145bp in spliced sequences were deleted, exon 44bp deletion (TGATCATGTCACAGTTCCAAATGCTCTTCCATCACCAACTGAAT) in the second exon.

*otpl-8*: 149-159bp in spliced sequences were deleted, 11bp deletion (ACCGACGACCT) in the second exon.

**Supplementary tables**

**Table S1. sgRNAs for creating each otopetrin mutant**

| **Genes** | **sgRNA** |
| --- | --- |
| *otpl-1* | TTTCTCACTGGTACTCGAGC |
| *otpl-2* | GAATAGCTTCCTTGCACAGA |
| *otpl-3* | GATATTGGCTTTCAGATGCG |
| *otpl-4* | TTGTAGTATATGCATTAGTC |
| *otpl-5* | GCTACTGAACCCGTCTGATG |
| *otpl-6* | TGTTAGGACAGTAACATCGC |
| *otpl-7* | AACTGTGACATGATCAAGTG |
| *otpl-8* | GCTCATGAATCCAAGGTCGT |

**Table S2. PCR primers used to genotype each otopetrin mutant**

| **Genes** | **Forward Primers** | **Reverse Primers** |
| --- | --- | --- |
| *otpl-1* | actgtagccttcgtggtttag | gcaactttgggactggttc |
| *otpl-2* | acactctctgctttatacgctc | caaatggaaagccacgtcca |
| *otpl-3* | acgcccgaaagtcatacttatc | gagccatgttccaagtacct |
| *otpl-4* | tttcagtgcatcgccaaag | gctcaatactccagagaacgat |
| *otpl-5* | agtttagtctagcgaccttgag | ccatcgacccgaagacttaa |
| *otpl-6* | ggacgcgggacaataagaag | gattctgattgccatgtcggag |
| *otpl-7* | tcctttgttcgggatacatctc | cttcctctccggcctttatg |
| *otpl-8* | gtttcgactagcggtttagaag | ccatgtcggtgagacttcaa |
